# Sequencing and analysis of the complete mitochondrial genome of *Datnioides campbelli* (Datnioididae)

**DOI:** 10.1080/23802359.2022.2143245

**Published:** 2022-11-11

**Authors:** Hong Zhou, Yexin Yang, Yi Liu, Hongmei Song, Xuejie Wang, Sudong Xia, Xidong Mu

**Affiliations:** a College of Fisheries, Tianjin Agricultural University, Tianjin, China; b Key Laboratory of Prevention and Control for Aquatic Invasive Alien Species, Ministry of Agriculture and Rural Affairs, Guangdong Modern Recreational Fisheries Engineering Technology Center, Pearl River Fisheries Research Institute, Chinese Academy of Fishery Sciences, Guangzhou, China

**Keywords:** New Guinea tiger fish, mitochondrial genome, next generation sequencing, *Datnioides campbelli*

## Abstract

In this study, the complete mitochondrial genome sequence of the New Guinea tiger fish *Datnioides campbelli* (Whitley 1938) (Lobotiformes: Datnioididae) was sequenced by next-generation sequencing method. The assembled mitochondrial genome consists of 13 protein-coding genes, 22 transfer RNA genes, and two ribosomal RNA genes, with a length of 16,416 bp. The total base composition of the mitogenome of *D. campbelli* was 29.31% for A, 29.02% for C, 15.14% for G and 26.54% for T. A phylogenetic tree based on 13 protein-coding genes (PCGs) provides important molecular data for further phylogeographic and evolutionary analysis of Lobotiformes.

## Introduction

*Datnioides campbelli,* commonly known as the New Guinea tiger fish, is an ornamental ray-finned fish belonging to the genus *Datnioides* (Family: Datnioididae, Order: Lobotiformes, Series: Eupercaria). The distribution of the New Guinea tiger fish is limited to the Gulf of Papua drainages and the coastal waters of New Guinea, occurring in brackish river mouths, coastal lagoons, and rivers above tidal influence (Roberts and Kottelat 1994). In this study, we focused on the New Guinea tiger fish due to its restricted distribution, high commercial value, and paucity of genetic research. This is the first report about the genomic study on *D. campbelli*. It provides a novel reference genome and important molecular data for Lobotiformes, which is a fundamental step toward resolving the phylogenetic relationships of the highly diverse ray-finned fish.

## Materials

**Figure 1.**
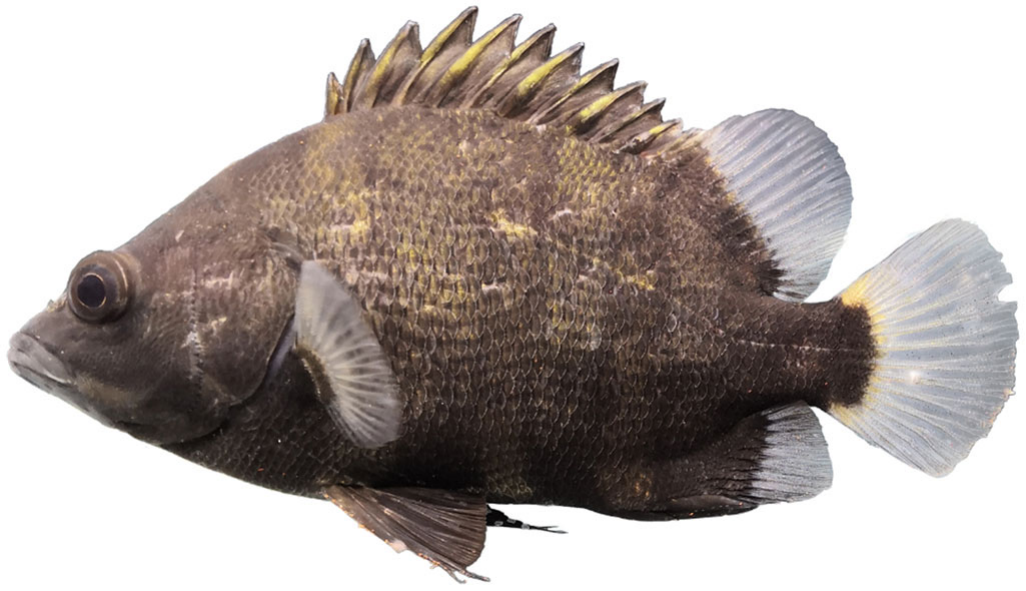
The reference picture of *Datnioides campbelli* in the present study (taken by Yexin Yang).

The specimen of *D. campbelli* (Figure 1) was collected from Guangzhou Lanhai Marine Technology Co., Ltd, Guangzhou city, Guangdong Province, China (latitude: 23°12′51″N, longitude: 113°28′6″E) and identified according to the morphological characters described in Roberts and Kottelat (1994). The muscle was preserved in 95% ethanol and stored at −80 °C. A specimen was deposited at the National Freshwater Genetic Resource Center in Guangzhou city, Guangdong Province in China (https://cafs-germplasm.app.msorg.cn/, Yexin Yang is the contact person yangyexin@prfri.ac.cn) under the voucher number DA-camp-1.

## Methods

Genomic DNA was extracted using a TIANamp Genomic DNA Kit (TIANGEN) and determined by a 0.8% agarose gel and Qubit® 2.0 fluorometer (Life Technologies, USA). The high-quality genomic DNA was used to prepare DNA library with an insert size of 350 bp using NEB Next® Ultra DNA Library Prep Kit for Illumina (NEB, USA) following manufacturer’s recommendations. 2 × 150 bp paired-end reads were generated on an Illumina Novaseq6000 platform using sequencing protocols provided by the manufacturer (Illumina, Inc., San Diego, CA). About 2.07 G raw data was generated. Raw reads from Illumina sequencing were subjected to adaptor trimming and filtering of low-quality reads by fastp v0.20.1(https://github.com/OpenGene/fastp) (Chen et al. 2018). The minimum length for reads after trimming was set to 150 nucleotides, and the quality threshold was set to Q20. The whole genome was assembled using SPAdes v.3.15.2 (http://cab.spbu.ru/software/spades/) (Lapidus et al. 2014) with ‘–plasmid’ option and kmer sizes 33, 55, 77, 99 and 127. The assembled contigs included a mixture of sequences from organellar and nuclear genomes. We identified mitochondrial contigs using similarity searches by BLASTN 2.13.0+ against NCBI Nucleotide collection (nt) database. MitoFish (http://mitofish.aori.u-tokyo.ac.jp/) (Iwasaki et al. 2013) was used to annotate the mitochondrial genome. The circular mitochondrial genome map of *D. campbelli* was visualized *via* CGView (Stothard and Wishart 2005). The study protocol was approved by the Laboratory Animal Ethics Committee of Pearl River Fisheries Research Institute, CAFS (number: LAEC-PRFRI-20201219).

The complete mitochondrial genome of *D. campbelli* was blasted against the GenBank database in NCBI, and 17 species highly similar to our *D. campbelli*, with Max score between 11,924 and 12,381, were selected to perform the phylogenetic analysis, including *Banjos banjos* (KT345965, Liu et al. 2016), *Caesio cuning* (KP874185, Zhan et al. 2017), *Datnioides polota* (MZ930122, unpublished), *Lutjanus argentimaculatus* (JN182927, Liao et al. 2013), *Lutjanus bengalensis* (FJ171339, Wang et al. 2010), *Lutjanus carponotatus* (MK092066, Kim et al. 2019), *Lutjanus fulgens* (MN398650, Afriyie et al. 2020), *Lutjanus kasmira* (FJ416614, Wang et al. 2010), *Lutjanus ophuysenii* (MZ042266, Sun et al. 2021), *Lutjanus peru* (KR362299, Bayona-Vásquez et al. 2017), *Lutjanus rivulatus* (AP006000, Yamanoue et al. 2007), *Lutjanus stellatus* (MW485060, unpublished), *Monodactylus argenteus* (AP009169, Yamanoue et al. 2007), *Prionurus biafraensis* (MN703417, Ludt et al. 2020), *Prionurus laticlavius* (MN703418, Ludt et al. 2020), *Pterocaesio digramma* (LC549803, Song et al. 2020), and *Stereolepis doederleini* (MT083886, Oh et al. 2021). Amongst, *Monodactylus argenteus* was selected as the outgroup. The alignment of concatenated 13 protein-coding genes (PCGs) from our *D. campbelli* together with the above 17 species were aligned using ClustalW in BioEdit (Hall et al. 2011) and then converted to Nexus file by PDGSpider 2.1.1.5 (Lischer abd Excoffier 2012), which was used as the import file for later phylogenetic analysis. A Bayesian phylogenetic tree was constructed based on 13 PCGs using MrBayes 3 (Ronquist and Huelsenbeck 2003). The specific settings were as follows: Four Metropolis-coupled Markov chain Monte Carlo (MCMC) analyses were run twice for 1,000,000 generations and sampled every 100 generations (mcmc ngen = 1,000,000; nchains = 4; temp = 0.01; samplefreq = 100; burnin = 2500) using the default general-time-reversible + gamma + invariants (GTR + G + I) model of sequence evolution and running until the standard deviation of the split frequencies was below 0.01. Then the first 25% of total trees were removed as burin-in, and the posterior probabilities (PP) was calculated by the remaining trees. The final tree was visualized in FigTree v1.4.4 (http://tree.bio.ed.ac.uk/).

## Results

**Figure 2.**
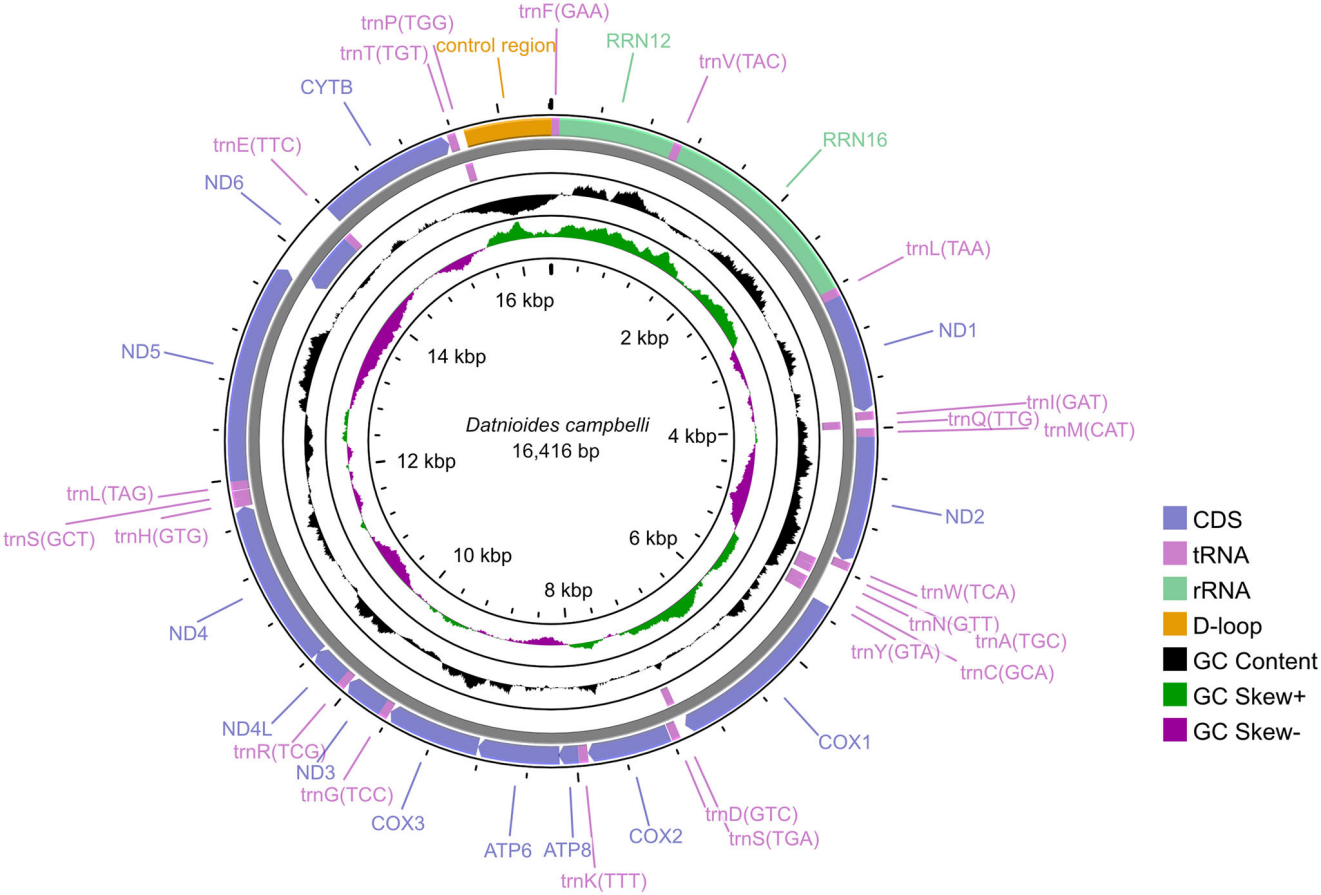
The circular mitochondrial genome map of *D. campbelli* in this study.

The complete mitochondrial genome of *D. campbelli* was 16,416 bp in length (GenBank: MZ930121), and included 13 protein-coding genes, 22 transfer RNAs, and two ribosomal RNA genes and a noncoding control region (D-loop) (Figure 2). The order of the mitogenome of *D. campbelli* was identical with that of *D. polota* (MZ930122, unpublished), and the overall sequence identity between *D. campbelli* and *D. polota* was up to 84.4%. The overall base composition of the complete mitogenome of *D. campbelli* was 29.31% for A, 29.02% for C, 15.14% for G, 26.54% for T.

**Figure 3.**
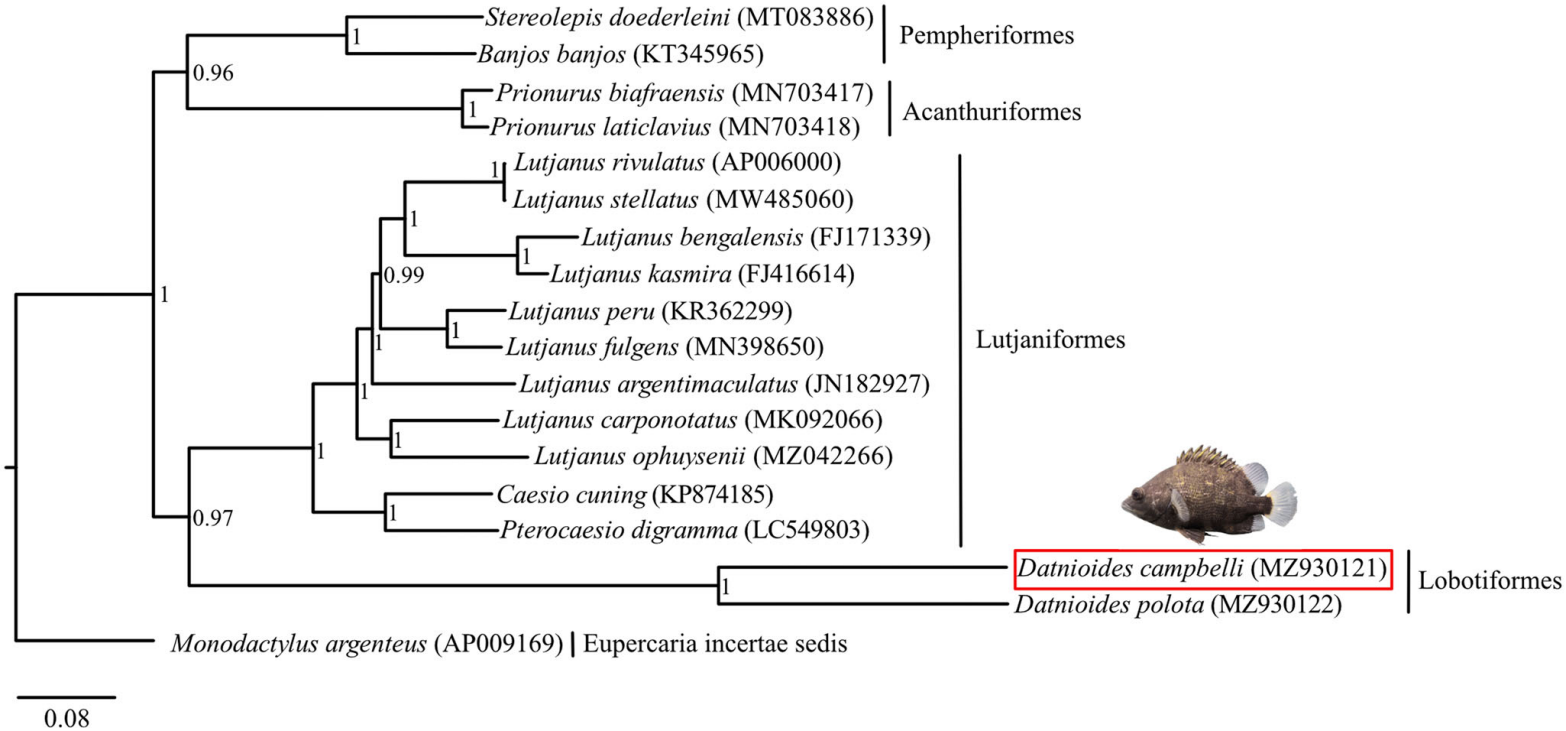
BI phylogenetic tree of 18 species in Eupercaria based on 13 protein-coding genes (PCGs). The numbers beside the nodes represent the Bayesian posterior probability. *D. campbelli* identified in this study (MZ930121) is labeled with a red box and upside is the picture of this species, taken by Yexin Yang.

The mitogenome of *D. campbelli* had typical vertebrate organization. All the 13 mitochondrial protein-coding genes shared the same start codon ATG, except for *COXI* (GTG start codon). The complete stop codon, TAA, was present in *ND2*, *COXI*, *ATP8*, *ATP6*, *COXIII*, *ND4*, *ND5* and *ND6*; TAG was present in *ND1*, *ND3*; and the incomplete stop codon ‘T––’ was found in *COXII*, *ND4* and *CYTB*. Of all protein-coding genes, the longest was the *ND5* gene (1,839 bp), and the shortest was the *ATP8* gene (168 bp).

Phylogenetic analysis based on 13 PCGs revealed that Lobotiformes was more closely related to Lutjaniformes, compared with Pempheriformes and Acanthuriformes (Figure 3).

## Discussion and conclusion

As the largest and most diverse group of vertebrates, ray-finned fishes account for half of all vertebrate species (Near et al. 2012; Hughes et al. 2018). In the present study, we first reported a novel mitochondrial genome of *D. campbelli* and the phylogeny inferred using the Bayesian method based on 13 PCGs showed that Lobotiformes is the sister lineage of Lutjaniformes, which is consistent with the previous study on the phylogeny of ray-finned fishes based on transcriptomic and genomic data (Hughes et al. 2018). Our study not only provides critical molecular data for further phylogeographic and evolutionary analysis of Lobotiformes, but also offers a basis for the later deep research on phylogenetic relationships in ray-finned fishes.

## Data Availability

The genome sequence data that support the findings of this study are openly available in GenBank of NCBI at (https://www.ncbi.nlm.nih.gov/genbank/) under accession number MZ930121. The associated BioProject, SRA, and Bio-Sample numbers are PRJNA787735, SRR17211917, and SAMN23839792, respectively.

## References

[CIT0001] Afriyie G, Wang Z, Dong Z, Ayisi Larbi C, Asiedu B, Guo Y. 2020. Complete mitochondrial genome and assembled DNA barcoding analysis of *Lutjanus fulgens* (Valenciennes, 1830) and its comparison with other Lutjanus species. Ecol Evol. 10(15):7971–7980. 32788954 10.1002/ece3.6542 PMC7417232

[CIT0003] Bayona-Vásquez NJ, Hernández-Álvarez CA, Glenn T, Domínguez-Domínguez O, Uribe-Alcocer M, Díaz-Jaimes P. 2017. Complete mitogenome sequences of the pacific red snapper (*Lutjanus peru*) and the spotted rose snapper (*Lutjanus gutattus*). Mitochondrial DNA Part A. 28(2):223–224. 10.3109/19401736.2015.1115851 26712305

[CIT0004] Chen S, Zhou Y, Chen Y, Gu J. 2018. fastp: an ultra-fast all-in-one FASTQ preprocessor. Bioinformatics. 34(17):i884–i890. 30423086 10.1093/bioinformatics/bty560 PMC6129281

[CIT0006] Hall T, Biosciences I, Carlsbad CJGBB. 2011. BioEdit: an important software for molecular biology. GERF Bulletin of Biosciences. 2(1):60–61.

[CIT0007] Hughes LC, Ortí G, Huang Y, Sun Y, Baldwin CC, Thompson AW, Arcila D, Betancur-R R, Li C, Becker L, et al. 2018. Comprehensive phylogeny of ray-finned fishes (Actinopterygii) based on transcriptomic and genomic data. Proc Natl Acad Sci USA. 115(24):6249–6254. 29760103 10.1073/pnas.1719358115 PMC6004478

[CIT0008] Iwasaki W, Fukunaga T, Isagozawa R, Yamada K, Maeda Y, Satoh TP, Sado T, Mabuchi K, Takeshima H, Miya M, et al. 2013. MitoFish and MitoAnnotator: a mitochondrial genome database of fish with an accurate and automatic annotation pipeline. Mol Biol Evol. 30(11):2531–2540. 23955518 10.1093/molbev/mst141 PMC3808866

[CIT0011] Kim G, Lee JH, Alam MJ, Lee SR, Andriyono S. 2019. Complete mitochondrial genome of Spanish flag snapper, *Lutjanus carponotatus* (Perciformes: lutjanidae). Mitochondrial DNA B. 4(1):568–569.

[CIT0012] Lapidus A, Antipov D, Bankevich A, Gurevich A, Korobeynikov A, Nurk S, Prjibelski A, Safonova Y, Vasilinetc I, Pevzner PA. 2014. New frontiers of genome assembly with SPAdes 3.0 [poster]. http://cab.spbu.ru/software/spades/.

[CIT0015] Liao J, Wang ZD, Guo YS, Liu CW. 2013. Complete mitochondrial sequence of *Lutjanus argentimaculatus* and Bayesian analysis. Marine Sciences. 37(1):62–69.

[CIT0016] Lischer HE, Excoffier L. 2012. PGDSpider: an automated data conversion tool for connecting population genetics and genomics programs. Bioinformatics. 28(2):298–299. 22110245 10.1093/bioinformatics/btr642

[CIT0017] Liu J, Jin W, Wu C. 2016. Complete mitochondrial genome of banjofish (*Banjos banjos*): genome characterization and phylogenetic analysis. Mitochondrial DNA A. 27(6):4433–4435. 10.3109/19401736.2015.1089559 26486169

[CIT0018] Ludt WB, Rocha LA, Chakrabarty P. 2020. The first complete mitochondrial genomes of sawtail surgeonfishes (Acanthuridae: *prionurus*). Mitochondrial Part B. 5(1):212–213. 10.1080/23802359.2019.1699465 PMC7748473 33366491

[CIT0019] Near TJ, Eytan RI, Dornburg A, Kuhn KL, Moore JA, Davis MP, Wainwright PC, Friedman M, Smith WL. 2012. Resolution of ray-finned fish phylogeny and timing of diversification. Proc Natl Acad Sci USA. 109(34):13698–13703. 22869754 10.1073/pnas.1206625109 PMC3427055

[CIT0020] Oh DJ, Lee JC, Ham YM, Jung YH. 2021. The mitochondrial genome of *Stereolepis doederleini* (Pempheriformes: polyprionidae) and mitogenomic phylogeny of Pempheriformes. Genetics and Molecular Biology. Genet Mol Biol. 44(1):e20200166. 33661273 10.1590/1678-4685-GMB-2020-0166 PMC7931504

[CIT0021] Roberts TR, Kottelat M. 1994. The Indo-Pacific tigerperches, with a new species from the Mekong basin (Pisces: coiidae). Ichthyol Explor Freshwaters. 5: 257–266.

[CIT0022] Ronquist F, Huelsenbeck JP. 2003. MrBayes 3: Bayesian phylogenetic inference under mixed models. Bioinformatics. 19(12):1572–1574. 12912839 10.1093/bioinformatics/btg180

[CIT0023] Song HY, Jung YH, Kim B, Choi YJ, Nguyen TV, Lee DS. 2020. Complete mitochondrial genome of the double-lined fusileer, *Pterocaesio digramma* (Perciformes, Caesionidae): mitogenome characterization and phylogenetic analysis. Mitochondrial DNA B Resour. 5(3):2617–2618. 33457882 10.1080/23802359.2020.1778575 PMC7782221

[CIT0024] Stothard P, Wishart DS. 2005. Circular genome visualization and exploration using CGView. Bioinformatics. 21(4):537–539. 15479716 10.1093/bioinformatics/bti054

[CIT0025] Sun P, Jiang Y, Yuan X, Zhang H. 2021. The complete mitochondrial genome of *Lutjanus ophuysenii* and phylogenetic analysis. Mitochondrial DNA B Resour. 6(8):2396–2397. 34345706 10.1080/23802359.2021.1951140 PMC8284153

[CIT0027] Wang ZD, Tan W, Guo YS, Liu L, Liu CW, Liu Y. 2010. Performance of mitogenomic coding regions within genus *Lutjanus* molecular phylogenetics. J Fish China. 34(6):656–664.

[CIT0028] Yamanoue Y, Miya M, Matsuura K, Yagishita N, Mabuchi K, Sakai H, Katoh M, Nishida M. 2007. Phylogenetic position of tetraodontiform fishes within the higher teleosts: Bayesian inferences based on 44 whole mitochondrial genome sequences. Mol Phylogenet Evol. 45(1):89–101. 17490896 10.1016/j.ympev.2007.03.008

[CIT0029] Zhan W, Chen RY, Shen KN, Xu DD, Hsiao CD, Lou B. 2017. Next-generation sequencing yields the complete mitochondrial genome of the Redbelly yellowtail fusilier, *Caesio cuning* (Teleostei: caesionidae). Mitochondrial DNA A. 28(1):125–126. 10.3109/19401736.2015.1111353 26709458

